# DualBranch-AMR: A Semi-Supervised AMR Method Based on Dual-Student Consistency Regularization with Dynamic Stability Evaluation

**DOI:** 10.3390/s25154553

**Published:** 2025-07-23

**Authors:** Jiankun Ma, Zhenxi Zhang, Linrun Zhang, Yu Li, Haoyue Tan, Xiaoran Shi, Feng Zhou

**Affiliations:** 1 The Key Laboratory of Electronic Information Countermeasure and Simulation Technology of Ministry of Education, Xidian University, Xi’an 710126, China; 23021211464@stu.xidian.edu.cn (J.M.); zhanglinrun@stu.xidian.edu.cn (L.Z.); yli1999@stu.xidian.edu.cn (Y.L.); 21021210621@stu.xidian.edu.cn (H.T.); xrshi@xidian.edu.cn (X.S.); 2 School of Aerospace Science and Technology, Xidian University, Xi’an 710126, China; fzhou@mail.xidian.edu.cn

**Keywords:** automatic modulation recognition, semi-supervised, dual-student model, dynamic stability, consistency constraints

## Abstract

Modulation recognition, as one of the key technologies in the field of wireless communications, holds significant importance in applications such as spectrum resource management, interference suppression, and cognitive radio. While deep learning has substantially improved the performance of Automatic Modulation Recognition (AMR), it heavily relies on large amounts of labeled data. Given the high annotation costs and privacy concerns, researching semi-supervised AMR methods that leverage readily available unlabeled data for training is of great significance. This study constructs a semi-supervised AMR method based on dual-student. Specifically, we first adopt a dual-branch co-training architecture to fully exploit unlabeled data and effectively learn deep feature representations. Then, we develop a dynamic stability evaluation module using strong and weak augmentation strategies to improve the accuracy of generated pseudo-labels. Finally, based on the dual-student semi-supervised framework and pseudo-label stability evaluation, we propose a stability-guided consistency regularization constraint method and conduct semi-supervised AMR model training. The experimental results demonstrate that the proposed DualBranch-AMR method significantly outperforms traditional supervised baseline approaches on benchmark datasets. With only 5% labeled data, it achieves a recognition accuracy of 55.84%, reaching over 90% of the performance of fully supervised training. This validates the superiority of the proposed method under semi-supervised conditions.

## 1. Introduction

With the number of IoT devices surpassing tens of billions, the continuous expansion of industrial internet applications, and the emergence of new concepts such as the metaverse, global wireless data traffic is experiencing explosive growth. Against this backdrop, communication systems face dual challenges of spectrum scarcity and quality-of-service demands, driving dynamic spectrum sharing and intelligent radio management technologies to become key research directions for 6G networks [1,2,3]. As a critical component of spectrum sensing systems, Automatic Modulation Recognition (AMR) analyzes the modulation characteristics of received signals, providing fundamental decision-making support for spectrum allocation optimization, interference suppression, and communication security [4,5]. Traditional AMR techniques can be broadly classified into two categories: feature extraction-based discriminative methods and probabilistic model-based generative approaches [6,7]. The former relies on expert-designed feature engineering, including higher-order statistics and spectral features, combined with traditional machine learning classifiers such as support vector machines [8] and decision trees [9]. The latter achieves classification by constructing likelihood functions. But traditional AMR methods rely on manual feature selection and shallow classifier design, whose performance faces significant degradation in complex propagation environments [10,11]. In recent years, deep learning has achieved breakthrough progress in the AMR field through its powerful capability of adaptive feature extraction, with its performance significantly surpassing traditional methods [12,13].

Although the deep AMR model based on supervised learning has achieved good results, its recognition effect depends on a large amount of annotated data requirements [14]. In real-world communication scenarios, the available labeled samples are often limited by multiple constraints. These include high costs of electromagnetic signal acquisition, timeliness requirements for spectrum monitoring, and privacy protection regulations. Meanwhile, unlabeled signals continue to be generated in large quantities within communication environments. Fully exploiting the implicit information hidden in these unlabeled signals can effectively enhance AMR performance without introducing additional annotation costs [15].

To reduce reliance on labeled samples, many semi-supervised learning methods have been employed to explore AMR, enhancing the model’s generalization ability by leveraging both limited labeled data and vast amounts of unlabeled data. Typical approaches, such as pseudo-labeling [16] and teacher–student models [17], have demonstrated potential under ideal channel conditions. However, existing methods still exhibit significant limitations when dealing with complex electromagnetic propagation environments. First, the two roles in the teacher–student framework remain tightly coupled, with this coupling intensifying progressively during training—a phenomenon that ultimately creates performance bottlenecks [17]. Second, complex time–frequency distortions (e.g., multipath fading, impulse noise) present in unlabeled signals lead to error accumulation in pseudo-label-based self-training approaches [16]. Third, conventional consistency regularization methods demonstrate limited capability in modeling spatiotemporal signal correlations, consequently failing to capture robust representations of modulation characteristics [18].

To address these challenges, this paper proposes a semi-supervised AMR method based on dual-student consistency regularization with dynamic stability evaluation (DualBranch-AMR). By jointly optimizing pseudo-label quality and feature representation robustness, it significantly improves modulation recognition performance under limited labeled data conditions. The main contributions of this paper include:

(1) To address the tight coupling issue in teacher–student models, we introduce dual-student networks with parameter heterogeneity. Through a stability interaction mechanism, we achieve full utilization of unlabeled data, effectively learn deep feature representations, and enhance the model’s semi-supervised performance.
(2) To enhance pseudo-label accuracy, we employ a dynamic stability evaluation module based on signal strong–weak augmentation to achieve confidence calibration of pseudo-labels, thereby reducing the mislabeling risk from noisy samples.
(3) To enhance the model’s capability in modeling spatiotemporal signal features, we propose a stability-guided consistency regularization method. This approach applies consistency constraints separately to strong–weak augmented data versions and dual-student stable outputs, enabling the model to effectively capture representational characteristics of signals across spatial–temporal dimensions.
(4) Extensive validation on public datasets RML2016.10A and RML2016.10B demonstrates the model’s superiority in recognition accuracy. The results show that with the same labeled sample resources, DualBranch-AMR outperforms existing semi-supervised SOTA models.

## 2. Related Work

### 2.1. Fully Supervised AMR

Current supervised learning-based AMR methods primarily advance technological development through two key dimensions: input feature optimization and network architecture innovation. At the feature engineering level, reference [19] broke through the traditional I/Q signal processing paradigm and innovatively adopted amplitude/phase features as inputs for Long Short-Term Memory networks, effectively capturing the temporal dependency characteristics of modulated signals. In [20], a Multi-Timing Constellation Diagram feature representation method was proposed, extracting the spatial distribution characteristics of constellation diagrams through multi-scale time windows. In terms of network architecture design, reference [21] systematically investigated the hyperparameter optimization strategies for Convolutional Neural Networks, revealing the impact mechanisms of network depth and filter dimensions on modulation classification performance. Reference [18] designed a novel complex-valued depthwise separable convolutional neural network for modeling the complex-valued features inherent in I/Q vectors. In [22], the Multi-Channel Long Short-Term Deep Neural Network (MCLDNN) model was innovatively designed with a multi-channel processing mechanism, enabling independent feature extraction and joint decision-making for I/Q signals, achieving state-of-the-art (SOTA) performance in public benchmark tests. However, these outstanding performance results achieved by the models are built upon massive labeled datasets, a stringent requirement that greatly limits their practical application value.

### 2.2. Semi-Supervised AMR

To reduce the reliance on labeled samples, numerous semi-supervised learning methods have been explored for AMR, which enhance model generalization by jointly leveraging limited labeled data and abundant unlabeled data [23,24]. In [25], a method based on Generative Adversarial Networks was introduced, where the generator synthesizes pseudo-samples with modulation class attributes, and the discriminator performs dual supervision by distinguishing sample authenticity and predicting modulation types. In [16], a Semi-Supervised Signal Recognition Convolutional Neural Network (SSRCNN) was developed, which employs multiple loss functions, including the Kullback–Leibler divergence and cross-entropy loss for unlabeled samples. Study [15] pioneered the use of self-supervised contrastive learning for representation learning of modulated signals, constructing positive and negative sample pairs by applying temporal transformations to unlabeled I/Q signals and learning discriminative signal representations during the pre-training phase, followed by fine-tuning the classifier on a small amount of labeled data. Reference [26] proposed a Convolutional Autoencoder structure with sparse representation; however, due to inherent limitations in the autoencoder architecture, it suffers from information loss, resulting in a lower performance ceiling. Zhang et al. [27] first pre-trained the model with a small set of labeled signals, then utilized the pre-trained model to generate pseudo-labels for unlabeled data to obtain an augmented dataset, and finally retrained the entire model using this dataset. In view of the above challenges, this study innovatively introduces a dynamic stability evaluation module to construct a robust pseudo-label screening mechanism through dynamic stability judgment, which can effectively alleviate the problem of error accumulation.

## 3. Materials and Methods

### 3.1. Signal Model and Problem Definition

This section outlines the signal model and provides an overview of the problem formulation for deep learning-based AMR.

#### 3.1.1. Signal Model

Consider a typical single-input single-output communication system, where the signal transmission process can be described as follows: the transmitted signal $s_{t}\left(t\right)$ propagates through a wireless channel to the receiver, during which the signal is affected by channel fading and additive noise. The mathematical model of the received signal can be expressed as:

(1) $$s_{r}\left(t\right)=s_{t}\left(t\right)*c\left(\tau ,t\right)+n\left(t\right)$$

where n(t) and c(τ,t) represent Gaussian noise and the fading channel, respectively. The received signal $s_{r}\left(t\right)$ undergoes a series of signal processing operations, including signal amplification and low-pass filtering, to eliminate out-of-band noise and enhance signal quality. Subsequently, the received signal is sampled at a specific sampling rate $f_{s}$, resulting in a complex-valued discrete signal sequence of length *N*:

(2) $$s_{r}=s_{r}^{I}+js_{r}^{Q}$$

where $j=\sqrt{-1}$, $s_{r}^{I}\in R^{L}$ as well as $s_{r}^{Q}\in R^{L}$ and refer to in-phase and quadrature components. In this paper, the complex-valued vector is treated as a two-dimensional real-valued vector, i.e.,

(3) $$x=\left[\begin{array}{c} s_{r}^{I}\\ s_{r}^{Q} \end{array}\right]$$

#### 3.1.2. Problem Formulation

Consider $D_{pt}=\left\{x_{pt}^{i}|i=0,1,\ldots ,N-1\right\}$ and $D_{ft}=\left\{\left(x_{ft}^{i},y^{i}\right)|i=0,1,\ldots ,M-1\right\}$ as the unlabeled dataset and the labeled dataset, respectively. Each signal $x_{ft}^{i}$ is associated with a label $y^{i}\in \left\{0,1,\ldots ,C-1\right\}$, where *C* represents the number of modulation types. Semi-supervised AMR primarily improves recognition accuracy by leveraging dataset $D_{pt}$ to assist in training the model.

In practical communication scenarios, acquiring large-scale labeled radio signal datasets is highly challenging. In contrast, obtaining unlabeled signals is relatively more convenient. Semi-supervised AMR not only reduces the economic and labor costs associated with labeling but is also more feasible to implement under conditions requiring privacy protection, security, and non-cooperation. Based on this, the core research objective of this paper is to design an efficient semi-supervised learning framework. This framework significantly enhances modulation recognition performance under semi-supervised conditions by deeply mining the latent feature information within unlabeled signals.

### 3.2. Our Method

In this section, we first present the overall architecture of the DualBranch-AMR method. Next, grounded in signal processing theory, we rigorously define the mathematical formulations for strong and weak augmentations. Building upon this foundation, we provide a comprehensive description of the dynamic stability evaluation module based on the strong–weak augmentation strategy. Finally, we introduce the stability-guided consistency regularization method, which optimizes the loss function through stability-based data selection to accomplish semi-supervised training.

#### 3.2.1. The Overall Architecture of DualBranch-AMR

**Figure 1:**
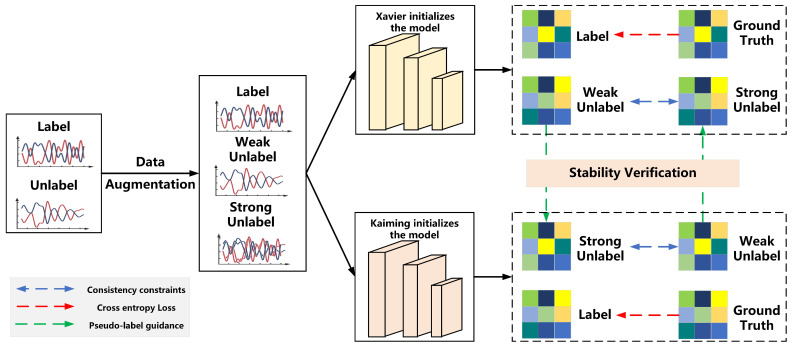
Overall architecture of DualBranch-AMR.

Figure 1 and Algorithm 1 outlines our proposed DualBranch-AMR model. First, unlabeled data undergoes strong and weak data augmentation, respectively. Then, we feed all data into two models with different initializations to obtain their corresponding output labels. The model then conducts stability evaluation on the unlabeled data outputs. When we determine that a data sample is stable for one model, we utilize its weakly augmented label scores to guide the strongly augmented label scores in the counterpart model, thereby facilitating collaborative learning between the models. Simultaneously, we impose consistency constraints on the strongly and weakly enhanced versions of unlabeled samples to fully exploit the spatiotemporal characteristics of the signal data. For labeled data, we apply the standard cross-entropy loss for supervised training.
**Algorithm 1** DualBranch-AMR**Require:** Labeled data $x_{ft}$, Unlabeled data $x_{pt}$, Initial model *f*  1: **for** each $x_{i}$ in $x_{pt}$ **do**  2:    $x^{weak}\leftarrow \mathrm{WeakAugment}\left(x_{i}\right)$ {Equation (7)}  3:    $x^{strong}\leftarrow \mathrm{StrongAugment}\left(x_{i}\right)$ {Equation (8)}  4: **end for**  5: $f_{1},f_{2}\leftarrow \mathrm{InitializeDualModels}\left(f\right)$ {Equations (9) and (10)}  6: **for** each epoch in all epoch **do**  7:    **for** each batch $\left(x_{ft},x^{weak},x^{strong}\right)$ **do**  8:        $\left(y_{ft-1}^{\prime },y_{\mathrm{weak}\text{-}1},y_{\mathrm{strong}\text{-}1}\right)\leftarrow f_{1}\left(x_{ft},x^{weak},x^{strong}\right)$ {Equation (11)}  9:        $\left(y_{ft-2}^{\prime },y_{\mathrm{weak}\text{-}2},y_{\mathrm{strong}\text{-}2}\right)\leftarrow f_{2}\left(x_{ft},x^{weak},x^{strong}\right)$ {Equation (12)}10:    **end for**11:    **for** each unlabeled sample *x* in $x_{pt}$ **do**12:        **for** each model *m* in $\left\{f_{1},f_{2}\right\}$ **do**13:            Determine if *x* is stable for *m* {Equations (13) and (14)}14:        **end for**15:        **if** both $f_{1}$ and $f_{2}$ find *x* stable **then**16:            Calculate loss *L* {Equations (15)–(18)}17:        **end if**18:    **end for**19:    Update $f_{1}$ and $f_{2}$ using total loss *L*20: **end for**

#### 3.2.2. Strong–Weak Augmentation for Signal Data

Data augmentation strategies play a crucial role in optimizing the performance of dual-student models, with their importance primarily reflected in the following two aspects. First, by generating diverse signal views, data augmentation enables the encoder to learn more robust and discriminative feature representations, thereby better adapting to the complex environmental variations in real-world communication scenarios. Second, well-designed data augmentation methods maintain label consistency between augmented samples and original samples. These methods preserve the semantic information of the signals while enhancing the model’s ability to learn generalized features that remain invariant to specific transformations.

This paper employs two types of signal data augmentation methods: phase offset (PO) and additive white Gaussian noise (AWGN). PO simulates phase distortion during channel propagation by introducing random phase variations, while AWGN simulates additive interference in real-world communication by adding random noise that follows a Gaussian distribution. The combined use of these two augmentation methods not only expands the distribution range of the training data but also enables the model to learn robust time–frequency feature representations. Additionally, the augmented input signals more closely resemble the characteristics of signals received in actual physical channels, thereby significantly enhancing the model’s adaptability and generalization performance in practical application scenarios. Since AWGN introduces greater data perturbation and disrupts the original data structure more significantly, we define it as the strong augmentation method, while PO serves as the weak augmentation approach.

(1) Weak Augmentation Method: PO
  In [15,28], the authors employ rotation as an augmentation method, i.e.
  (4) $$\begin{array}{cc} PO(x) & =R\cdot x=\left[\begin{array}{c} \cos\varphi_{0}\;\;-\sin\varphi_{0}\\ \sin\varphi_{0}\;\;\;\;\;\cos\varphi_{0} \end{array}\right]\left[\begin{array}{c} s_{r}^{I}\\ s_{r}^{Q} \end{array}\right]\\ & =\left[\begin{array}{c} s_{r}^{I}\cos\varphi_{0}-s_{r}^{Q}\sin\varphi_{0}\\ s_{r}^{I}\sin\varphi_{0}+s_{r}^{Q}\cos\varphi_{0} \end{array}\right] \end{array}$$
  where *R* is the rotation matrix and $\varphi_{0}$ is the phase offset. The first and second terms of the above formula represent the real and imaginary parts of the augmented version, respectively. Therefore, we can further simplify it by transforming it as follows:
  (5) $$\begin{array}{cc} & \left(s_{r}^{I}\cos\varphi_{0}-s_{r}^{Q}\sin\varphi_{0}\right)+j\left(s_{r}^{I}\sin\varphi_{0}+s_{r}^{Q}\cos\varphi_{0}\right)\\ & =\left(s_{r}^{I}+js_{r}^{Q}\right)\cdot \left(\cos\varphi_{0}+j\sin\varphi_{0}\right)\\ & =Xe^{j\varphi_{0}} \end{array}$$
(2) Strong Augmentation Method: AWGN
  As a classic noise model, it is widely used to characterize random interference caused by the motion of electrons at the receiver front end. By introducing random noise that follows a Gaussian distribution into the signal, AWGN effectively simulates the statistical properties of random processes such as thermal noise in electronic devices.
  (6) AWGN(x)=x+N
  where *N* represents Gaussian white noise with a certain signal-to-noise ratio (SNR) relative to *x*.

#### 3.2.3. Dynamic Stability Assessment Module Based on Strong–Weak Augmentation Strategy

We introduce the concept of Stable Samples [17], which must satisfy the following two conditions: First, according to the smoothness assumption, a small perturbation should not affect the prediction of this sample, meaning the model should be smooth in the vicinity of this sample. Second, the prediction of this sample should be far from the decision boundary, indicating that the sample has a high probability for the predicted label. The mathematical formulation is as follows:

**Definition** **1.** *Given a constant ξ∈[0,1), a dataset $D\in R^{m}$ that satisfies the smoothness assumption, and a model $f:D\to {\left[0,1\right]}^{n}$, for all x∈D satisfying ${∥f\left(x\right)∥}_{1}=1$, if:*

(1) *For any $\bar{x}\in D$ and x that are close in the feature space, their predicted labels are the same;*
(2) *x satisfies the inequality: ${∥f\left(x\right)∥}_{\infty }>\xi$.*

*Then, x is considered a stable sample relative to f.*

*When an unlabeled sample is a stable sample, we consider the label generated by the model for that sample as a valid label, which is then used to guide semi-supervised training.*

*Based on the concept of stable samples, a dynamic stability assessment module using the strong–weak augmentation strategy is defined as follows: If a signal sample is stable for the model, the model’s predictions for the strongly and weakly augmented versions of the signal should be consistent. Additionally, the predicted label probabilities for the augmented samples should be high. The specific mathematical formulation is as follows:*

**Definition** **2.** *Given a variable ξ, a signal dataset $D\in R^{m}$ and a model $f:D\to {\left[0,1\right]}^{n}$, for all x∈D satisfying ${∥f\left(x\right)∥}_{1}=1$, where $x_{strong}$ and $x_{weak}$ represent the strongly augmented and weakly augmented versions of sample x, respectively, if:*

(1) *The unique heat labels output by the same model for $x_{strong}$ and $x_{weak}$ should be consistent.*
(2) *$x_{strong}$ and $x_{weak}$ satisfy the inequalities: $\left\{{∥f\left(x_{\mathrm{weak}\;}\right)∥}_{\infty }>\xi \right\}\&\left\{{∥f\left(x_{\mathrm{strong}\;}\right)∥}_{\infty }>\xi \right\}$.*

*Then, x is considered a stable sample relative to f. Parameter ξ is a variable that continuously changes during model training (see Section 3.3).*

#### 3.2.4. Stability-Guided Consistency Regularization Approach

To enable dual models to extract robust spatiotemporal features from diverse data perspectives while achieving mutual guidance, we propose a stability-guided consistency regularization method. First, we apply weak and strong data augmentations to the unlabeled dataset. Weak augmentation aims to slightly modify the data while preserving its key features, whereas strong augmentation explores diverse representations by significantly transforming the data. For any unlabeled data point $x_{pt}$, its weakly augmented version $x_{weak}$ and strongly augmented version $x_{strong}$ are generated.

(7) $$x_{\mathrm{weak}\;}=PO\left(x_{pt}\right)$$

(8) $$x_{\mathrm{strong}\;}=AWGN\left(x_{pt}\right)$$

Subsequently, we initialize the model *f* using Kaiming initialization [29] and Xavier initialization [30] to obtain models $f_{1}$ and $f_{2}$, respectively.

(9) $$f_{1}=\mathrm{Xavier}\left(f\right)$$

(10) $$f_{2}=\mathrm{Kaiming}\left(f\right)$$

The labeled data $x_{ft}$, along with the strongly augmented unlabeled data $x_{\mathrm{strong}\;}$ and weakly augmented unlabeled data $x_{\mathrm{weak}\;}$, are input into models $f_{1}$ and $f_{2}$, respectively, to obtain the corresponding model prediction scores.

(11) $$\left\{\begin{array}{c} y_{ft-1}^{\prime }=f_{1}\left(x_{ft}\right)\\ y_{\mathrm{weak}\text{-}1}=f_{1}\left(x_{\mathrm{weak}}\right)\\ y_{\mathrm{strong}\text{-}1}=f_{1}\left(x_{\mathrm{strong}}\right) \end{array}\right.$$

(12) $$\left\{\begin{array}{c} y_{ft-2}^{\prime }=f_{2}\left(x_{ft}\right)\\ y_{\mathrm{weak}-2}=f_{2}\left(x_{\mathrm{weak}}\right)\\ y_{\mathrm{strong}-2}=f_{2}\left(x_{\mathrm{strong}}\right) \end{array}\right.$$

Next, we perform stability assessment on the strongly and weakly augmented signal samples:

(13) $$D_{pt}^{i1}=\left\{y_{\mathrm{strong}\text{-}1\;}^{i1}=y_{\mathrm{weak}\;-1}^{i1}\right\}\&\left(\left\{{∥f_{1}\left(x_{\mathrm{strong}\;}^{i1}\right)∥}_{\infty }>\xi \right\}\&\left\{{∥f_{1}\left(x_{\mathrm{weak}\;}^{i1}\right)∥}_{\infty }>\xi \right\}\right)$$

(14) $$D_{pt}^{i2}=\left\{y_{\mathrm{strong}\text{-}2\;2}^{i2}=y_{\mathrm{weak}\text{-}2\;}^{i2}\right\}\&\left(\left\{{∥f_{2}\left(x_{\mathrm{strong}\;}^{i2}\right)∥}_{\infty }>\xi \right\}\&\left\{{∥f_{2}\left(x_{\mathrm{weak}\;}^{i2}\right)∥}_{\infty }>\xi \right\}\right)$$

where $D_{pt}^{i1}$ and $D_{pt}^{i2}$ represent the sets of stable samples relative to model $f_{1}$ and model $f_{2}$, respectively. ξ represents the logical AND, indicating that the sample needs to satisfy both sides of the equation to be recognized as a stable sample.

To enable the model to learn feature representations that are robust to channel distortion and noise interference, we impose consistency constraints between strongly and weakly augmented signal samples.

(15) $$L_{\mathrm{mse}1}={∥y_{\mathrm{weak}\;-1}-y_{\mathrm{strong}\;-1}∥}_{2}+{∥y_{\mathrm{weak}\;-2}-y_{\mathrm{strong}\text{-}\;2}∥}_{2}$$

To enable mutual guidance between the dual student models, we enforce consistency constraints on their outputs for stable samples. Specifically, we minimize the Mean Squared Error between the predictions of weakly augmented inputs from one student and strongly augmented inputs from the other student:

(16) $$L_{\mathrm{mse}2}={∥y_{\mathrm{weak}\text{-}1\;}^{i1}-y_{\mathrm{strong}\;-2}^{i1}∥}_{2}+{∥y_{\mathrm{weak}\text{-}\;2}^{i2}-y_{\mathrm{strong}\;-1}^{i2}∥}_{2}$$

Here, $y_{\mathrm{weak}\text{-}1\;}^{i1}$ represents the results of weakly augmented stable samples passing through Model 1, $y_{\mathrm{strong}\;-2}^{i1}$ represents the results of strongly augmented stable samples passing through Model 2, $y_{\mathrm{weak}\text{-}\;2}^{i2}$ represents the results of weakly augmented stable samples passing through Model 2, and $y_{\mathrm{strong}\;-1}^{i2}$ represents the results of strongly augmented stable samples passing through Model 1.

For labeled samples, we employ the standard cross-entropy loss to ensure correct classification:

(17) $$L_{cel}=-\sum_{i=1}^{C}y_{ft}\log\left(y_{ft-1}^{\prime }\right)-\sum_{i=1}^{C}y_{ft}\log\left(y_{ft-2}^{\prime }\right)$$

where *C* is the number of classes, and $y_{ft}$ represents the true labels of the labeled data. The overall training objective combines both losses with a balancing hyperparameter λ:

(18) $$L=L_{cel}+\lambda \left(L_{mse1}+L_{mse2}\right)$$

where λ is a hyperparameter that balances the constraints, enabling the model to benefit from both labeled data and consistency across students.

### 3.3. Datasets and Experimental Settings

We conducted the experiments on two public datasets, RML2016.10A and RML2016.10B [31,32], which DeepSIG generated using GNU Radio (3.7). Each signal in the datasets consists of complex time-domain IQ samples and was generated in a hsarsh simulated propagation environment. This environment incorporates factors such as AWGN, multipath fading, sampling rate offsets, and center frequency offsets to simulate real-world conditions.

(1) RML2016.10A: This dataset comprises 220,000 samples with signal-to-noise ratios ranging from −20 dB to 18 dB. Each sample has a signal length of 128, and there are a total of 11 modulation types: WBFM, AM-DSB, AM-SSB, BPSK, CPFSK, GFSK, 4-PAM, 16-QAM, 64-QAM, QPSK, and 8-PSK.
(2) RML2016.10B: This dataset comprises 1.2 million samples with signal-to-noise ratios ranging from −20 dB to 18 dB. Each sample has a signal length of 128, and there are a total of 10 modulation types: WBFM, AM-DSB, BPSK, CPFSK, GFSK, 4-PAM, 16-QAM, 64-QAM, QPSK, and 8-PSK.

In all experiments, we use Adam [33] as the optimizer. During the semi-supervised training phase, we train the model for 200 epochs with a learning rate of 0.0003 and a batch size of 256 to avoid local optima. The hyperparameter is set to 5. Training is stopped early if the validation loss does not show improvement for more than 20 epochs. We conduct the experiments in a Windows environment and use the open-source PyTorch (0.3.0) framework for training and testing. The GPU utilized is an NVIDIA GeForce RTX 4060 (8 GB).

## 4. Results

### 4.1. Evaluation of DualBranch-AMR

The experiments utilize all data from RML2016.10A and RML2016.10B for training. The dataset is split into training, testing, and validation sets in a ratio of 7:1:2. To ensure the fairness and reliability of the experiments, an equal number of samples for each modulation type is selected at every SNR.

To systematically evaluate the impact of labeled data quantity and semi-supervised learning on AMR performance, we conduct experiments on RML2016.10A and RML2016.10B using stratified random sampling to select labeled subsets from the original training data while preserving class distributions. We test six regimes (N = 2, 5, 10, 20, 50, 100 labeled samples per class), covering 0.05–2.44% (RML2016.10B) and 0.2–10% (RML2016.10A) of total samples. For labeled samples, the strength and weakness enhancement method is also used to augment the dataset. To evaluate the model’s classification performance, we primarily use recognition accuracy—the proportion of correctly predicted samples among all test instances.

**Figure 2:**
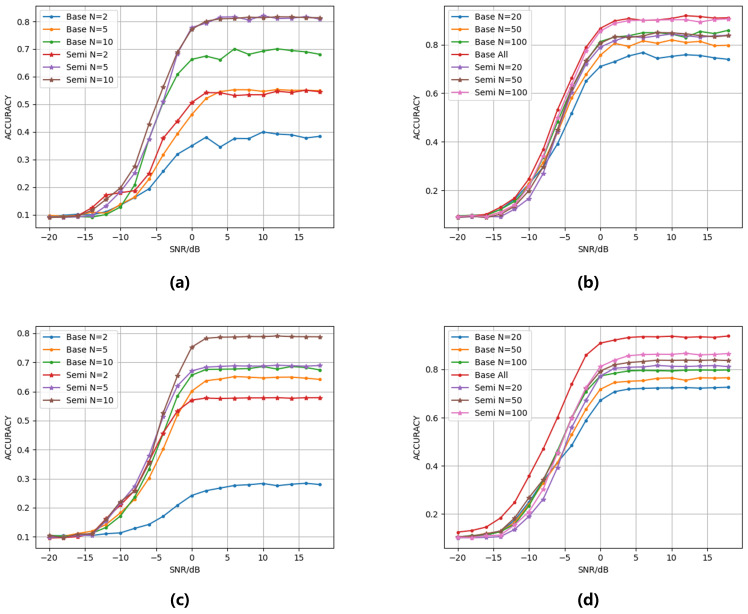
The model’s accuracy under varying signal-to-noise ratios (SNRs) and different numbers of labeled samples: (**a**) RML2016.10A, N = 2, 5, 10. (**b**) RML2016.10A, N = 20, 50, 100, all. (**c**) RML2016.10B, N = 2, 5, 10. (**d**) RML2016.10B, N = 20, 50, 100, all. The notation “base” indicates the model trained without semi-supervised learning, while “semi” denotes the model trained with semi-supervised learning, and “all” represents fully supervised learning.

As shown in Figure 2, the classification accuracy of both datasets shows a significant increasing trend with the number of labeled samples. Moreover, with the same number of labeled samples, the accuracy of the semi-supervised model is always higher than that of the base model without semi-supervised learning. When the number of labeled samples N = 100, our method achieves a peak accuracy of 89.91% on the RML2016.10A dataset, reducing the gap to only 1.99% compared to the fully supervised benchmark performance of 91.90% in the existing literature [22]. The experimental results demonstrate that DualBranch-AMR can effectively enhance model performance in semi-supervised scenarios by leveraging mutual guidance from unlabeled data. Notably, a marked performance discrepancy emerges between semi-supervised and fully supervised approaches on the RML2016.10B dataset. This discrepancy primarily arises from the dataset’s larger scale relative to RML2016.10A. With only N = 100 labeled samples, representing a mere 2.44 percent of the total dataset, the limited annotated data proves inadequate for effectively learning robust features across the complete data distribution.

This paper systematically evaluates the effectiveness of the DualBranch-AMR approach across varying annotation scales (N = 2 to N = 100) on RML2016.10A/B datasets through comparative experiments. As shown in Table 1, the DualBranch-AMR method demonstrates significant advantages across all annotation levels. In extreme low-resource scenarios with only two labeled samples, the semi-supervised strategy shows absolute improvements of 26.72% to 36.93% for RML2016.10A and of 20.11% to 40.78% for RML2016.10B. These results confirm the method’s exceptional robustness when facing severely limited annotations. With merely five labeled samples, the model achieves 52.93% recognition accuracy, attaining 86.8% of the 61.12% accuracy achieved by the fully supervised benchmark. The experimental results demonstrate that DualBranch-AMR effectively alleviates the strong dependency of supervised learning on labeled data by mining implicit structures in unlabeled data. This approach provides an effective paradigm for model optimization in annotation-constrained real-world scenarios.

**Figure 3:**
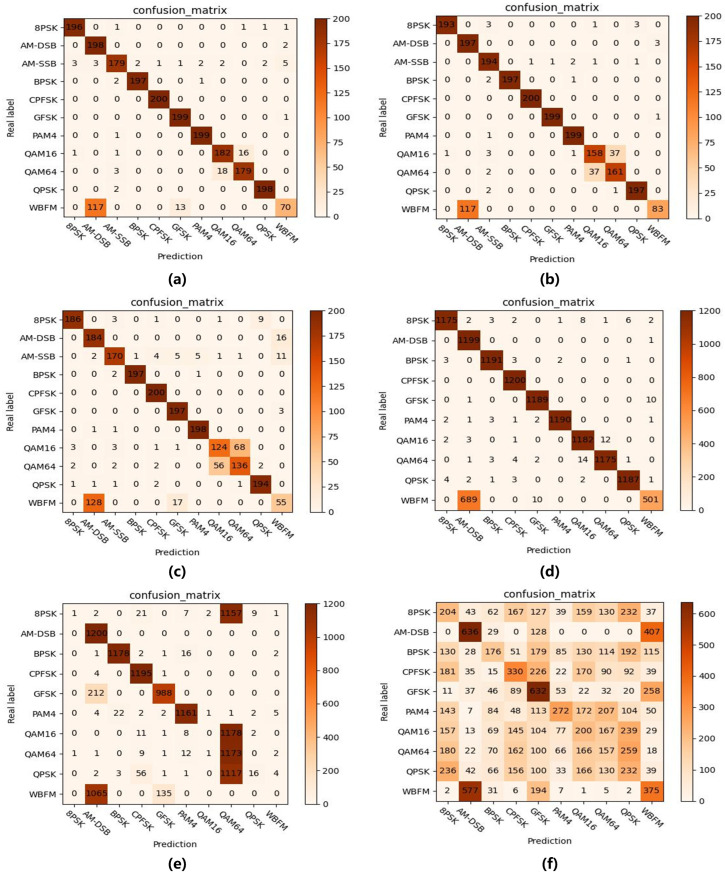
Recognition confusion matrices at SNR = 4 dB: (**a**) RML2016.10A, N = all, (**b**) RML2016.10A, N = 100 (semi), (**c**) RML2016.10A, N = 100 (base), (**d**) RML2016.10B, N = all, (**e**) RML2016.10B, N = 100 (semi), (**f**) RML2016.10B, N = 100 (base). The notation “base” indicates the model trained without semi-supervised learning, while “semi” denotes the model trained with semi-supervised learning, and “all” represents fully supervised learning.

Additionally, the confusion matrix for our method’s recognition is shown in Figure 3. On the RML2016.10A dataset, under fully supervised conditions, only 8.60% of QAM data resulted in confusion. When sample labels were limited, the model without semi-supervised methods confused 32.29% of QAM data, whereas our semi-supervised approach reduced this to just 18.82%. The results demonstrate that our semi-supervised method can assist the model in recognizing QAM data by uncovering latent features from unlabeled samples, thereby reducing the confusion rate among similar modulation types. Meanwhile, due to silent periods in the audio signals, all models struggled to accurately identify WBFM signals [21]. On the RML2016.10B dataset, when labeled data is extremely scarce (N = 2), the model without semi-supervised learning fails to achieve proper recognition, demonstrating only limited identification capability for AM-DSB and GFSK. In contrast, our semi-supervised approach can effectively recognize multiple modulation types, such as AM-DSB and BPSK. These results further validate that our method can significantly enhance model recognition performance by leveraging abundant unlabeled data, even under severely label-deficient conditions.

**Figure 4:**
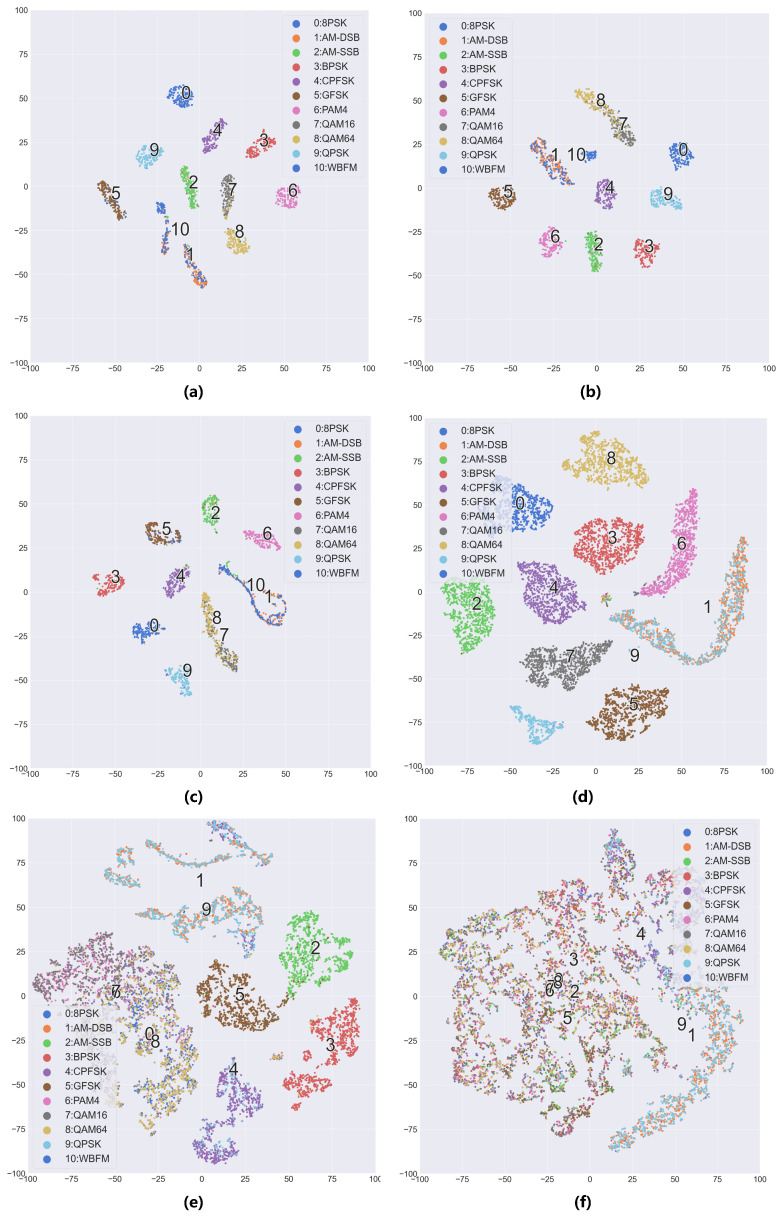
TSNE feature visualization chart when the SNR is 4 dB: (**a**) RML2016.10A, N = all, (**b**) RML2016.10A, N = 100 (semi), (**c**) RML2016.10A, N = 100 (base), (**d**) RML2016.10B, N = all, (**e**) RML2016.10B, N = 100 (semi), (**f**) RML2016.10B, N = 100 (base). The notation “base” indicates the model trained without semi-supervised learning, while “semi” denotes the model trained with semi-supervised learning, and “all” represents fully supervised learning.

Figure 4 displays the t-SNE feature visualization of the models. On the RML2016.10A dataset, the fully supervised model exhibits the clearest class boundaries, forming compact and well-separated clusters for all modulation types except WBFM and AM-DSB. When labeled samples are limited, the model without semi-supervised learning shows overlapping QAM feature clusters, while the semi-supervised model maintains QAM clusters with only boundary-level overlaps, consistent with the confusion matrix results. Similarly, on the RML2016.10B dataset with extremely scarce labeled samples, the model without semi-supervised fails to form well-separated clusters, whereas the semi-supervised model achieves feature separation for certain modulation types.

### 4.2. Comparison with Other Semi-Supervised Methods

**Figure 5:**
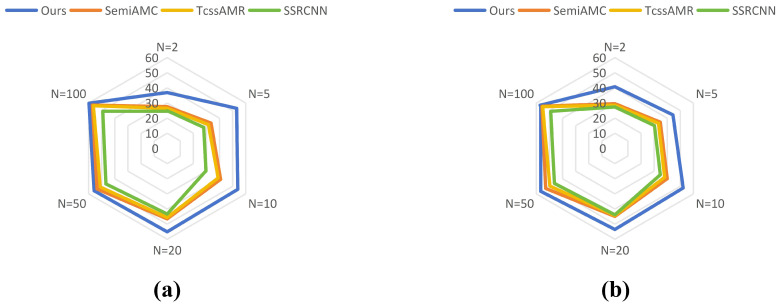
Overall performance of recognition accuracy between DualBranch-AMR and semi-supervised AMR methods under different numbers of labeled samples on RML2016.10A dataset (**a**) and RML2018.01A (**b**).

**Figure 6:**
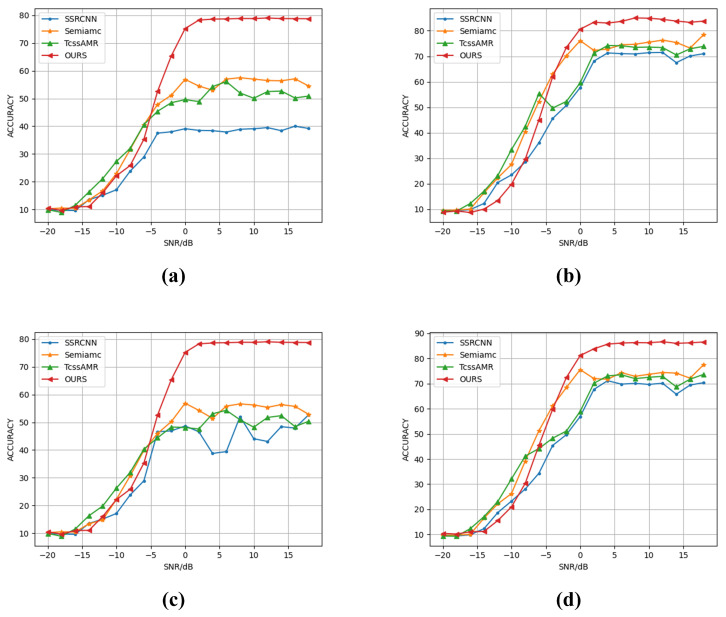
Accuracy of various semi-supervised methods under different SNRs and varying numbers of labeled samples on RML2016.10A and RML2016.10B datasets: (**a**) RML2016.10A, N = 10, (**b**) RML2016.10A, N = 50, (**c**) RML2016.10B, N = 10, (**d**) RML2016.10B, N = 50.

As Figure 5 and Figure 6, experimental results on the RML2016.10A dataset demonstrate that our proposed method exhibits significant advantages over current state-of-the-art semi-supervised approaches across all labeled sample quantities. At N = 2, it attains 36.93% classification accuracy, outperforming SemiAMC (27.60%), TcssAMR (26.50%), and SSRCNN (24.93%) by 9.33%, 10.43%, and 12.00%, respectively. This substantial improvement highlights our model’s exceptional capability in extracting robust features under extreme label scarcity. As the labeled data increases to 100, our method maintains its leading position with 59.75% accuracy, surpassing the second competitor SemiAMC by 2.55%. These results collectively validate that our framework effectively leverages information from unlabeled data, successfully addressing the performance limitation imposed by annotation costs in semi-supervised learning scenarios.

Experiments on the RML2016.10B dataset further confirm the proposed method’s generalization capability and cross-domain adaptability. As Figure 5 and Figure 6, our approach demonstrates systematic superiority across all annotation scales, with performance gains following interpretable patterns consistent with the RML2016.10A findings. Specifically, at N = 2, our method achieves 40.78% accuracy, substantially leading baseline models (SemiAMC: 29.62%, TcssAMR: 28.53%, SSRCNN: 27.41%), which underscores its profound exploitation of limited supervisory signals. When increasing to N = 10 labeled samples, the method reaches 52.17% accuracy, establishing a 12.23% absolute advantage over the suboptimal SemiAMC (39.94%). Even at N = 100, the method preserves a 1.4% performance lead. Cross-dataset analysis proves the method’s universal advantages under limited supervision, offering novel technical solutions to the fundamental trade-off between annotation resources and model performance in practical applications.

### 4.3. Ablation Experiment

**Figure 7:**
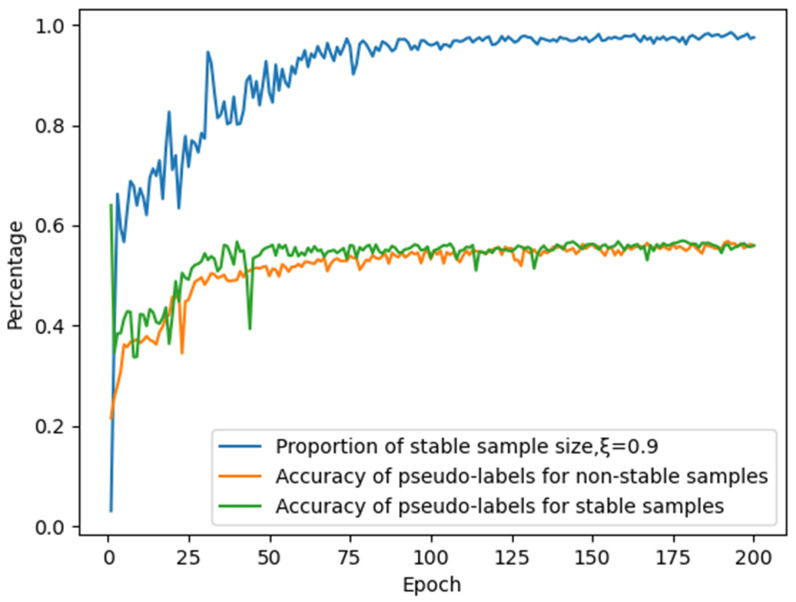
Impact of the stability module on pseudo-label accuracy and quantity.

For the RML2016.10A dataset, we trained our model using only 10% of the unlabeled data with parameter ξ set to 0.9. As shown in Figure 7, the incorporation of our proposed dynamic stability evaluation module based on strong–weak augmentation strategies significantly improves the accuracy of generated pseudo-labels. However, the results reveal that during initial training stages, while pseudo-label accuracy remains high, the number of samples satisfying stability criteria is limited, resulting in substantial underutilization of available unlabeled data samples.

**Figure 8:**
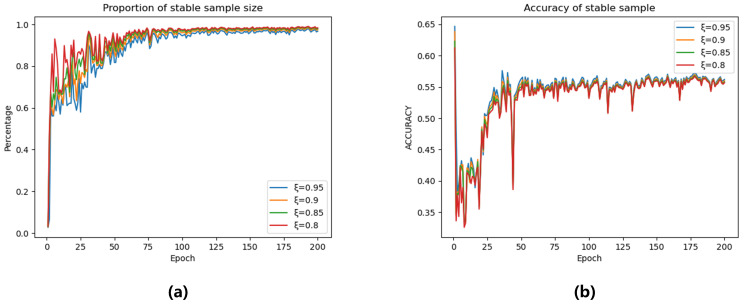
The impact of parameter *ξ* on (**a**) the number of stable samples and (**b**) pseudo-label accuracy of stable samples across training epochs.

To further investigate the impact of the pseudo-label generation module, we varied the hyperparameter ξ. As Figure 8, as ξ increases, the number of stable samples progressively decreases. However, larger ξ values consistently yield higher pseudo-label accuracy for stable samples. While elevated ξ values provide improved pseudo-label precision, they also lead to significant underutilization of unlabeled data. To balance this trade-off, we design ξ as a dynamically increasing function of training epochs, formulated as:

(19) $$\xi =\left\{\begin{array}{c} 0.8+0.15\times i/50,i\leq 50\\ 0.95,i>50 \end{array}\right.$$

where *i* denotes the current training epoch.

To validate the effectiveness of core modules in the proposed framework, we conducted ablation studies on the RML2016.10A dataset with 100 labeled samples. As presented in Table 2, the experiments adopted a layer-wise addition strategy, starting from the MCLDNN baseline model and progressively incorporating (1) the Dual-Student Network, (2) the Dynamic Stability Evaluation Module, and (3) the Stability-Guided Consistency Regularization Method.

With only the dual-student architecture, the model achieved 58.58% accuracy—a 1.79% improvement over the non-semi-supervised baseline, confirming the effectiveness of divergent optimization paths through differential perturbations. Introducing the dynamic stability evaluation module increased accuracy to 59.41%, demonstrating that higher pseudo-label accuracy effectively facilitates model training. The complete model attains 59.75% accuracy, marking a 0.34% enhancement over the preceding stage. This improvement verifies that enforcing consistency constraints between strongly and weakly augmented versions of samples effectively strengthens the model’s capacity to understand and extract discriminative time–frequency features.

## 5. Conclusions

This paper proposes a semi-supervised AMR method based on dual-student consistency regularization with dynamic stability evaluation, aiming to address the challenge of scarce labeled data in complex electromagnetic environments. The proposed approach employs a dual-student architecture, dynamic stability evaluation module, and stability-guided consistency regularization method to achieve significant performance improvements under limited labeled data conditions. Experimental results demonstrate that DualBranch-AMR exhibits excellent generalization capabilities on both the RML2016.10A and RML2016.10B datasets, with its robustness advantages being particularly pronounced in low signal-to-noise ratio scenarios. Specifically, in extreme low-annotation scenarios, the proposed method achieves a relative accuracy improvement of over 10%, validating its ability to deeply exploit limited labeled signals. Furthermore, ablation experiments confirm the effectiveness of each module, with the dual-student network, the dynamic stability evaluation module, and stability-guided consistency regularization method contributing 1.79%, 0.83%, and 0.34% accuracy improvements, respectively. Our method not only provides a new technical pathway for semi-supervised AMR but also offers theoretical and methodological support for data-efficient learning in other signal processing tasks. Future work will explore model optimization in more complex channel environments and deployment validation in practical communication systems.

## Figures and Tables

**Table 1:** Accuracy comparison of models with and without semi-supervised learning on RML2016.10A and RML2016.10B datasets.

RML2016.10A	Base (MCLDNN)	Ours	△ (↑%)	RML2016.10B	Base (MCLDNN)	Ours	△ (↑%)
N = 100	56.79%	59.75%	2.96%	N = 100	54.40%	57.10%	2.70%
N = 50	53.87%	55.84%	1.97%	N = 50	51.57%	56.64%	5.07%
N = 20	50.13%	55.07%	4.94%	N = 20	49.30%	53.52%	4.22%
N = 10	45.80%	53.96%	8.16%	N = 10	45.51%	52.17%	6.66%
N = 5	35.66%	52.93%	17.27%	N = 5	43.15%	47.10%	3.95%
N = 2	26.72%	36.93%	10.21%	N = 2	20.11%	40.78%	20.67%
supervised learning	61.12%			supervised learning	62.51%		

**Table 2:** Analysis of each active ingredient.

Dual Student	Dynamic Stability	Consistency Regularization	Accuracy
Network	Evaluation Module	for Strong-Weak Data Augmentation	
×	×	×	56.79%
✓	×	×	58.58%
✓	✓	×	59.41%
✓	✓	✓	59.75%

## Data Availability

The data comes from publicly available datasets [31,32].

## References

[1] He J., Huang S., Yang Z., Yu K., Huan H., Feng Z. (2023). Channel-agnostic radio frequency fingerprint identification using spectral quotient constellation errors. IEEE Trans. Wirel. Commun..

[2] Qiu K., Zheng S., Zhang L., Lou C., Yang X. (2023). DeepSIG: A hybrid heterogeneous deep learning framework for radio signal classification. IEEE Trans. Wirel. Commun..

[3] Zhang D., Ding W., Zhang B., Xie C., Li H., Liu C., Han J. (2018). Automatic modulation classification based on deep learning for unmanned aerial vehicles. Sensors.

[4] Ding G., Wang J., Wu Q., Yao Y.D., Song F., Tsiftsis T.A. (2015). Cellular-base-station-assisted device-to-device communications in TV white space. IEEE J. Sel. Areas Commun..

[5] Ke Z., Vikalo H. (2021). Real-time radio technology and modulation classification via an LSTM auto-encoder. IEEE Trans. Wirel. Commun..

[6] Huang S., Chai L., Li Z., Zhang D., Yao Y., Zhang Y., Feng Z. (2019). Automatic modulation classification using compressive convolutional neural network. IEEE Access.

[7] Dobre O.A., Abdi A., Bar-Ness Y., Su W. (2007). Survey of automatic modulation classification techniques: Classical approaches and new trends. IET Commun..

[8] Park C.S., Choi J.H., Nah S.P., Jang W., Kim D.Y. Automatic modulation recognition of digital signals using wavelet features and SVM. Proceedings of the 2008 10th International Conference on Advanced Communication Technology.

[9] Yuan J., Zhao-Yang Z., Pei-Liang Q. Modulation classification of communication signals. Proceedings of the IEEE MILCOM 2004. Military Communications Conference.

[10] Wu H.C., Saquib M., Yun Z. (2008). Novel automatic modulation classification using cumulant features for communications via multipath channels. IEEE Trans. Wirel. Commun..

[11] Zhu D., Mathews V.J., Detienne D.H. (2018). A likelihood-based algorithm for blind identification of QAM and PSK signals. IEEE Trans. Wirel. Commun..

[12] Zhang J., Wang T., Feng Z., Yang S. (2023). Toward the automatic modulation classification with adaptive wavelet network. IEEE Trans. Cogn. Commun. Netw..

[13] Liu Y., Yan X., Hao X., Yi G., Huang D. (2023). Automatic modulation recognition of radiation source signals based on data rearrangement and the 2D FFT. Remote Sens..

[14] Xu J., Luo C., Parr G., Luo Y. (2022). Quadruplet convolution neural network with C3 loss (C3-QCNN) for signal classification. IEEE Trans. Instrum. Meas..

[15] Liu D., Wang P., Wang T., Abdelzaher T. Self-contrastive learning based semi-supervised radio modulation classification. Proceedings of the MILCOM 2021-2021 IEEE Military Communications Conference.

[16] Dong Y., Jiang X., Cheng L., Shi Q. (2021). SSRCNN: A semi-supervised learning framework for signal recognition. IEEE Trans. Cogn. Commun. Netw..

[17] Ke Z., Wang D., Yan Q., Ren J., Lau R.W.H. Dual student: Breaking the limits of the teacher in semi-supervised learning. Proceedings of the 2019 IEEE/CVF International Conference on Computer Vision.

[18] Xiao C., Yang S., Feng Z. (2023). Complex-valued depthwise separable convolutional neural network for automatic modulation classification. IEEE Trans. Instrum. Meas..

[19] Rajendran S., Meert W., Giustiniano D., Lenders V., Pollin S. (2018). Deep learning models for wireless signal classification with distributed low-cost spectrum sensors. IEEE Trans. Cogn. Commun. Netw..

[20] Mao Y., Dong Y.Y., Sun T., Rao X., Dong C.X. (2021). Attentive siamese networks for automatic modulation classification based on multitiming constellation diagrams. IEEE Trans. Neural Netw. Learn. Syst..

[21] Tekbıyık K., Ekti A.R., Görçin A., Kurt G.K., Keçecı C. Robust and fast automatic modulation classification with CNN under multipath fading channels. Proceedings of the 2020 IEEE 91st Vehicular Technology Conference (VTC2020-Spring).

[22] Xu J., Luo C., Parr G., Luo Y. (2020). A spatiotemporal multi-channel learning framework for automatic modulation recognition. IEEE Wirel. Commun. Lett..

[23] Xiao C., Yang S., Feng Z., Jiao L. (2023). Mclhn: Towards automatic modulation classification via masked contrastive learning with hard negatives. IEEE Trans. Wirel. Commun..

[24] Li Y., Shi X., Tan H., Zhang Z., Yang X., Zhou F. (2024). Multi-Representation Domain Attentive Contrastive Learning Based Unsupervised Automatic Modulation Recognition. Nat. Commun..

[25] Li M., Liu G., Li S., Wu Y. Radio classify generative adversarial networks: A semi-supervised method for modulation recognition. Proceedings of the 2018 IEEE 18th International Conference on Communication Technology.

[26] O’Shea T.J., West N., Vondal M., Clancy T.C. Semi-supervised radio signal identification. Proceedings of the 2017 19th International Conference on Advanced Communication Technology.

[27] Zhang Y., Zhao Z. (2021). Limited data spectrum sensing based on semi-supervised deep neural network. IEEE Access.

[28] Davaslioglu K., Boztaş S., Ertem M.C., Sagduyu Y.E., Ayanoglu E. (2022). Self-supervised RF signal representation learning for NextG signal classification with deep learning. IEEE Wirel. Commun. Lett..

[29] He K., Zhang X., Ren S., Sun J. Delving deep into rectifiers: Surpassing human-level performance on imagenet classification. Proceedings of the IEEE International Conference on Computer Vision.

[30] Glorot X., Bengio Y. Understanding the difficulty of training deep feedforward neural networks. Proceedings of the Thirteenth International Conference on Artificial Intelligence and Statistics.

[31] O’Shea T.J., Corgan J., Clancy T.C. Convolutional radio modulation recognition networks. Proceedings of the Engineering Applications of Neural Networks, Proceedings of the 17th International Conference, Aberdeen, UK, 2–5 September 2016.

[32] O’Shea T.J., Roy T., Clancy T.C. (2018). Over-the-air deep learning based radio signal classification. IEEE J. Sel. Top. Signal Process..

[33] Kingma D.P., Ba J. (2024). Adam: A method for stochastic optimization. arXiv.

